# A stratification model of hepatocellular carcinoma based on expression profiles of cells in the tumor microenvironment

**DOI:** 10.1186/s12885-022-09647-5

**Published:** 2022-06-04

**Authors:** Chunting Zeng, Linmeng Zhang, Chanhua Luo, Chen Yang, Xiaowen Huang, Linfeng Fan, Jiarong Li, Fengsheng Chen, Zelong Luo

**Affiliations:** 1 Cancer Center, Nanfang Hospital, Southern Medical University, 510315 Guangzhou, China; 2 Cancer Center, Zhujiang Hospital, Southern Medical University, 510315 Guangzhou, China; 3 Cancer Center, Integrated Hospital of Traditional Chinese Medicine, Southern Medical University, 510315 Guangzhou, China; 4 State Key Laboratory of Oncogenes and Related Genes, Shanghai Cancer Institute, Renji Hospital, Shanghai Jiao Tong University School of Medicine, Shanghai, China; 5 Digestive system department, The Sixth Affiliated Hospital, South China University of Technology, Guangzhou, 510315 China; 6 Key Laboratory of Gastroenterology and Hepatology, Ministry of Health, Division of Gastroenterology and Hepatology, Renji Hospital, Shanghai Cancer Institute, Shanghai Institute of Digestive Disease, Shanghai Jiao Tong University School of Medicine, Shanghai, China; 7 Digestive system department, The Fifth Affiliated Hospital Sun Yat-sen University, Zhuhai, China

**Keywords:** Hepatocellular carcinoma, Immunotherapy, Tumor microenvironment, Tumor treatment, Stratification model

## Abstract

**Background:**

A malignancy of the liver, hepatocellular carcinoma (HCC) is among the most common and second-leading causes of cancer-related deaths worldwide. A reliable prognosis model for guidance in choosing HCC therapies has yet to be established.

**Methods:**

A consensus clustering approach was used to determine the number of immune clusters in the Cancer Genome Atlas and Liver Cancer-RIKEN, JP (LIRI_JP) datasets. The differentially expressed genes (DEGs) among these groups were identified based on RNA sequencing data. Then, to identify hub genes among signature genes, a co-expression network was constructed. The prognostic value and clinical characteristics of the immune clusters were also explored. Finally, the potential key genes for the immune clusters were determined.

**Results:**

After conducting survival and correlation analyses of the DEGs, three immune clusters (C1, C2, and C3) were identified. Patients in C2 showed the longest survival time with the greatest abundance of tumor microenvironment (TME) cell populations. MGene mutations in Ffibroblast growth factor-19 *(FGF19)* and catenin (cadherin-associated protein),β1*(CTNNB1)* were mostly observed in C2 and C3, respectively. The signature genes of C1, C2, and C3 were primarily enriched in 5, 23, and 26 pathways, respectively.

**Conclusions:**

This study sought to construct an immune-stratification model for the prognosis of HCC by dividing the expression profiles of patients from public datasets into three clusters and discovering the unique molecular characteristics of each. This stratification model provides insights into the immune and clinical characteristics of HCC subtypes, which is beneficial for the prognosis of HCC.

**Supplementary Information:**

The online version contains supplementary material available at 10.1186/s12885-022-09647-5.

## Background

Despite the treatments and diagnostic methods that have emerged over time for hepatocellular carcinoma (HCC), the long-term survival of HCC patients remains poor [1]. Currently, Since the development of molecular biology and molecular immunology, immunotherapy has gained considerable attention in cancer treatment. Safety and efficacy of nivolumab, an anti-PD-1 immune checkpoint inhibitor was previously evaluated in a clinical trial of advanced HCC patients and the outcome was promising [2]. Providing novel insight into the prognosis of and treatment options for HCC was the purpose of the current study by establishing an immune-stratification model by classifying the HCC tumor microenvironment (TME) into three subtypes.

## Methods

### Data collection and preprocessing

We obtained transcriptional profile and mutation spectrum of TCGA (The Cancer Genome Atlas) cohort from the TCGA website (https://portal.gdc.cancer.gov/repository). Survival information of the 377 HCC samples in TCGA cohort were downloaded from the TCGA Pan-cancer Clinical Data Resource. 21 samples without survival data were excluded. We downloaded raw RNA-Seq read count data and clinical data of the LIRI_JP cohort from Liver Cancer-RIKEN,JP (LIRI_JP) dataset of the International Cancer Genome Consortium (https://dcc.icgc.org/projects/LIRI-JP). In addition, we normalized all raw counts data into transcript per million (TPM) for downstream analyses.

### Immune score, stromal score, and microenvironment cell population (MCP)-counter

The Estimation of Stromal and Immune Cells in Malignant Tumour Tissues Using Expression Data (ESTIMATE) algorithm was used to calculate immune and stromal scores. Based on transcriptomic data, MCP-counter evaluated the absolute abundance of eight immune and two non-immune stromal cell populations by using the MCPcounter package of the R software program (R Foundation for Statistical Computing, Vienna, Austria).

### Consensus clustering for TME cell populations

Based on the NbClust and ConsensuClusterPlus R packages, the number of clusters was determined for the TCGA and LIRI_JP cohorts using a consensus clustering algorithm. A 1000-times repetition of the process ensured the stability of classification.

### Signature genes associated with different TME subtypes

According to the TME cell population–infiltrating patterns, patients were divided into three groups—namely, C1, C2, and C3. The expression differences between groups were characterized by log2 fold-change. Subsequently, pathway enrichment analysis was performed using the Enrichr (http://amp.pharm.mssm.edu/Enrichr/) database and the top five pathways with values of *p* < 0.05 were added into bubble plots.

### Identification of hub genes among signature genes of different subtypes

The WGCNA package in R was applied to calculate the correlation coefficient based on the RNA sequencing data (expression values were log-transformed). Then, correlation data were imported into the Cytoscape version 3.0 software program (Cytoscape Consortium, San Diego, CA, USA; https://cytoscape.org/) to construct the gene co-expression network. Co-expression network edges were specified to have correlation coefficients > 0.7 for C2 and C3.

### Statistical analysis

Normality was verifed using Kolmogorov–Smirnov and Shapiro–Wilk tests (*p* > 0.05 signifies the variables that were normally distributed). The cutoff values of absolute abundance of TME cell populations and gene expression were evaluated using the survminer R package. A univariate Cox proportional hazards regression model was established to calculate the hazard ratios (HRs) for univariate analyses. All statistical and computational analyses were conducted by R programming (https://www.r-project.org/) and GraphPad Prism version 8.0 (GraphPad, San Diego, CA, USA; https://www.graphpad.com/), and a two-tailed value of *p* < 0.05 was considered to be statistically significant.

## Results

### Landscape of TME cell populations in HCC

**Fig. 1:**
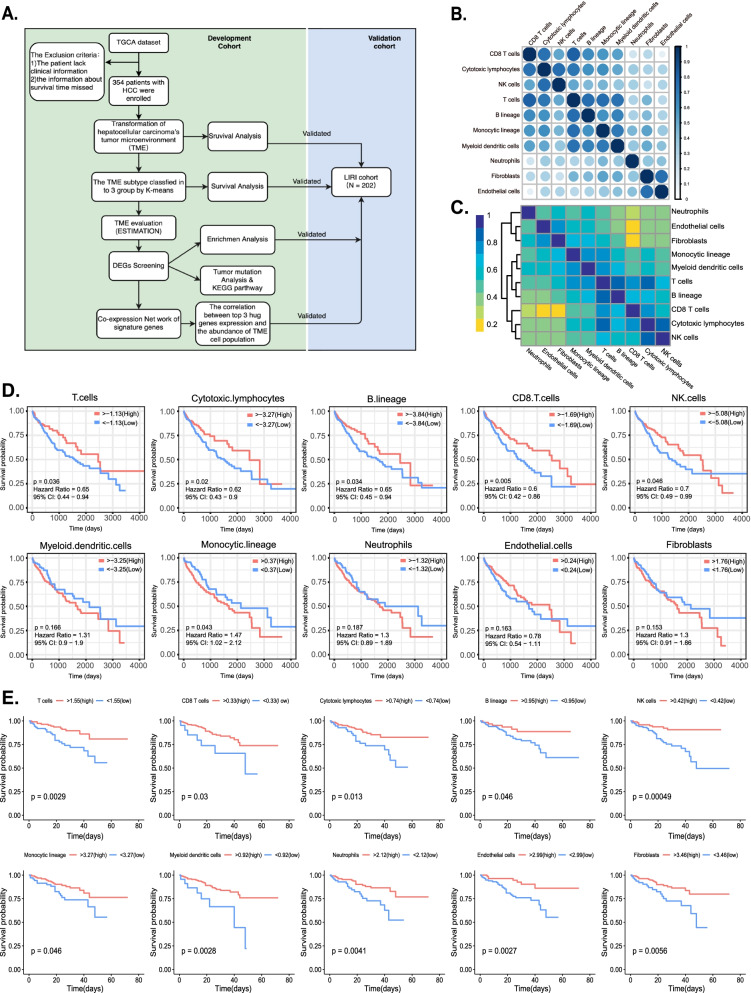
The landscape of immune infiltration in hepatocellular carcinoma (HCC). **a** Flowchart depicting the study design. Correlation heatmap of the tumor microenvironment (TME) cell populations in the Cancer Genome Atlas (TCGA) cohort **b** and Liver Cancer-RIKEN,JP (LIRI_JP) cohort (**c**). Kaplan–Meier curves of patients distinguished by the optimal cutoff of absolute abundance of TME cell populations in the TCGA cohort **d** and LIRI_JP cohort **e**. Log-rank tests were used to determine whether differences between groups are statistically significant

Our study was systematically described with a flowchart (Fig. 1a). Totals of 345 and 202 patients from the TCGA and LIRI_JP datasets, respectively, were enrolled in this study. Based on the gene-expression matrix of several distinct experimental groups, the MCP-counter was used to determine the abundance of different TME cell populations. Subsequently, a correlation heatmap of TME cell populations in the TCGA cohort (Fig. 1b) was constructed to investigate the relationship between the eight immune and two non-immune stromal cell populations. It has been shown that there is a significant correlation between the eight immune cell types and two stromal cell types in the heatmap, and the same result has also been confirmed in the LIRI_JP cohort (Fig. 1c). Based on the abundance of TME cells in the patients, two categories were created: high or low abundance based on the optimal cutoff value determined by the cutpoint function in the survminer package. The results of survival analysis indicated that high infiltration by T-cells (HR = 0.65, 95% confidence interval [CI] = 0.44–0.94, *p* = 0.036), cytotoxic lymphocytes (HR = 0.62, 95% CI = 0.43–0.90, *p* = 0.020), a B lineage (HR = 0.65, 95% CI = 0.45–0.94, *p* = 0.034), CD8^+^T-cells (HR = 0.60, 95% CI = 0.42–0.86, *p* = 0.005), and natural killer (NK) cells (HR = 0.70, 95% CI = 0.49–0.99, *p* = 0.046) correlated with a significantly favorable prognosis, whereas a monocytic lineage (HR = 1.47, 95% CI = 1.02–2.12, *p* = 0.043) was associated with a poorer outcome. In the LIRI_JP group, similar results were also observed in that the prognosis was improved in conjunction with the high infiltration of T-cells, cytotoxic lymphocytes, B lineage, CD8^+^T-cells, and NK cells. Different from the TCGA cohort, however, LIRI_JP patients with a high infiltration of myeloid dendritic cells, neutrophils, fibroblasts, and endothelial cells also had favorable outcomes. The Kaplan–Meier curve for the above-identified cell populations and that for the rest of the TCGA and LIRI_JP cohorts are shown in Fig. 1d and e, respectively.

### Identification of TME subtypes associated with prognostic subtypes

**Fig. 2:**
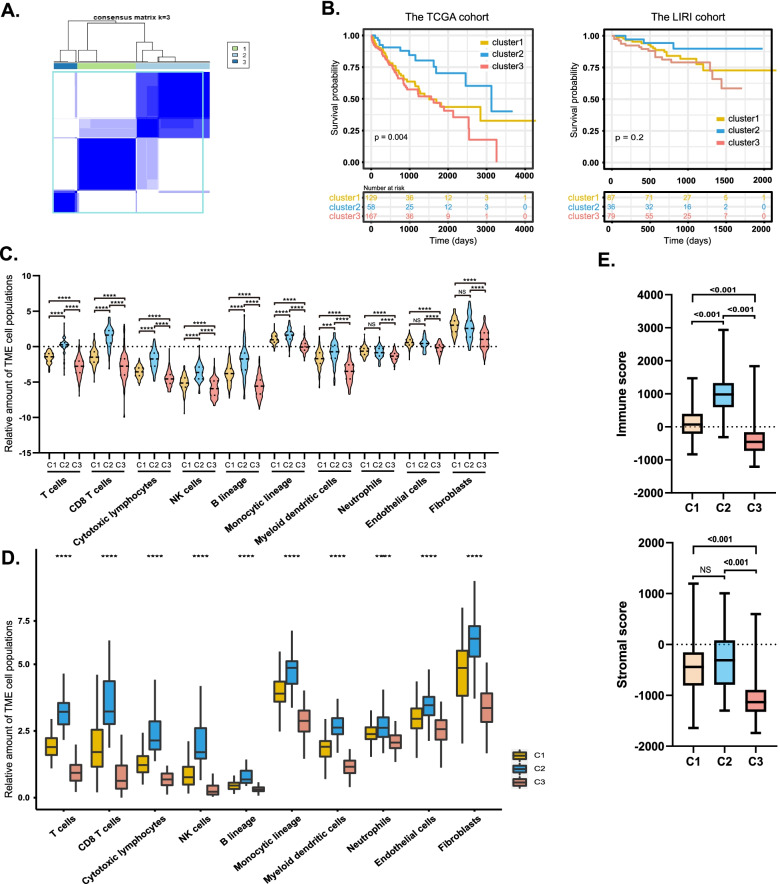
Unsupervised clustering of tumor microenvironment (TME) cell populations in the Cancer Genome Atlas (TCGA) cohort. **a** Consensus matrix heatmap from K-means consensus clustering identified 3 different clusters—namely, C1, C2, and C3. **b** Kaplan-Meier curves showed the overall survival rates for patients with hepatocellular carcinoma (HCC) in TCGA and Liver Cancer-RIKEN,JP (LIRI_JP) cohorts. Patients were divided by different TME subtypes. Violin plot of the absolute abundance of TME cell populations distinguished by different TME subtypes in the TCGA cohort **(c)** and LIRI_JP cohort **d**. *Abbreviation:* ns, no significance. *p < 0.05, **p < 0.01, ***p < 0.001, ***p < 0.0001). A log-rank test was used to determine the statistical significance of differences. **e** Immune and stromal scores were determined using ESTIMATION for patients of 3 TME subtypes in the TCGA cohort. Medians, interquartile ranges, and minimum/maximum are shown in boxplots, and the differences between each group were compared by using the Mann–Whitney *U* test

According to the optimal cluster number (k = 3, Fig. 2a) determined by the NbClust and ConsensusClusterPlus packages, patients in the TCGA cohort were classified into three groups (C1, C2, and C3). The details of K-means consensus clustering are shown in Fig. S1. A significant prognostic difference was observed (*p* = 0.004, Fig. 2b) among these three groups, for C2, the median survival time (MST) is longer (*n* = 58, MST = 3125 days) than C1 (*n* = 129, MST = 1508 days) or C3 (*n* = 167, MST = 1560 days). Another HCC dataset from the LIRI_JP cohort was adopted to validate its prognostic value, though there was no statistical difference of the median survival time for the clusters in the LIRI-JP cohort, the similar trend that C2 has a longer median survival time for C2 than C1 or C3 can be observed (*p* = 0.2, Fig. 2b). To confirm the difference between the three subtype classifications, the absolute abundance of TME cell populations for the TCGA cohort (Fig. 2c) was estimated, and results revealed that seven types of TME cells were involved (T-cells, CD8+ T-cells, NK cells, cytotoxic lymphocytes, myeloid cells, and monocytic cells; all *p* < 0.001) had the highest abundance in C2, while C3 had the lowest abundance of these seven TME cell types. Additionally, As for neutrophils (*p* = 0.114), endothelial cells (*p* = 0.191), and fibroblasts (*p* = 0.268) there were no significant differences between C1 and C2. A similar result was also validated in the LIRI_JP cohort (Fig. 2d) in that the amounts of T-cells, CD8^+^ T-cells, cytotoxic lymphocytes, NK cells, B-cell lineage cells, and monocytic lineage cells (all *p* < 0.001) were highest in C2 and lowest in C3, respectively. Additionally, ESTIMATION is used to determine immune and stromal scores, and significant differences between the three clusters were found (Fig. 2e). This provides powerful evidence for the validity of the subtype classification.

### TME subtypes in the TCGA cohort: clinical characteristics

**Fig. 3:**
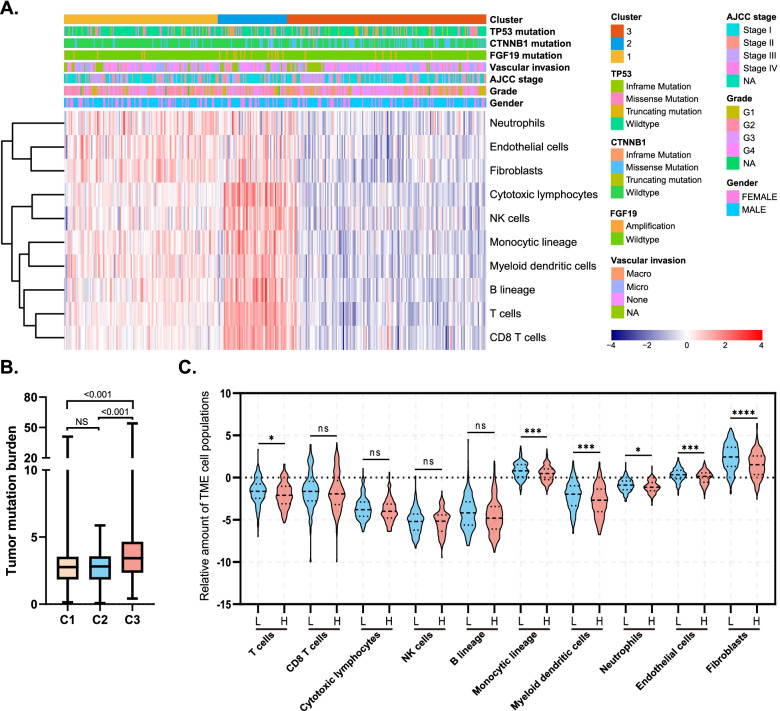
Clinical characteristics of different TME subtypes. **a** Heatmap describing the absolute abundance of tumor microenvironment (TME) cell populations in TME subtypes C1, C2, and C3. Group information; mutation status of *TP53*, *CTNNB1*, and *FGF19*; vascular invasion; American Joint Committee on Cancer stage; histological grade; and gender are shown as patient annotations. **b** Boxplot of the tumor mutation burden in three TME subtypes. The differences were compared by using the Mann–Whitney *U* test. **c** Violin plot of the absolute abundance of TME cell populations between the low-TMB and high-TMB groups distinguished by median value. The differences between each subtype were compared using Student’s *t*-test

Clinical characteristics of different subtypes in the TCGA cohort, including age at diagnosis, gender, TNM stage, histologic grade, vascular invasion, and genetic changes (tumor protein p53*[TP53]*,*CTNNB1,*and *FGF19*) are shown in Fig. 3a and Table 1. According to the results of the analysis, *CTNNB1* and *FGF19* had different genetic alterations among the three TME types. For example, the frequency of *CTNNB1* gene mutations was higher in C3 (37.7%) than C1 (13.2%) or C2 (24.1%), and the frequency of *FGF19* genetic alterations was higher in C2 (20.7%) than C1 (3.9%) or C3 (4.2%). In addition, C3 had a higher number of somatic mutations per megabase (TMB) than C1 or C2. (*p* < 0.001), while between C1 and C2, TMB did not differ significantly (*p* = 0.990, Fig. 3b). Subsequently, a low-TMB and high-TMB group of patients was formed based on the median value of TMB, and there were significant differences in the amounts of T-cells (*p* = 0.022), monocytic lineage cells (*p* < 0.001), myeloid dendritic cells (*p* < 0.001), neutrophils (*p* = 0.037), endothelial cells (*p* < 0.001), and fibroblasts (*p* < 0.001) between the two groups (Fig. 3c).

**Table 1:** Clinical characteristics of patients of different subtypes in the Cancer Genome Atlas cohort

Variable	C1	C2	C3	*p* value
Gender				0.561
Female	47	19	51	
Male	82	39	116	
Age (years)				0.371
< 60	56	31	77	
≥ 60	73	26	90	
Grade				0.330
I	17	9	25	
II	69	25	75	
III	39	20	58	
IV	1	3	8	
AJCC stage				0.896
I	55	29	82	
II	31	14	36	
III	27	13	42	
IV	3	0	2	
Vascular invasion				0.752
None	71	32	93	
Micro	33	11	45	
Macro	6	3	5	
*TP53*				0.447
Mutant	35	21	52	
Wild-type	94	37	115	
*CTNNB1*				< 0.001
Mutant	17	14	63	
Wild-type	112	44	104	
*FGF19*				< 0.001
Amplification	5	12	7	
Wild-type	124	46	160	

### Clinical characteristics of TME subtypes in the LIRI_JP cohort

The clinical characteristics of different subtypes in the LIRI cohort, as well as age, gender, and stage of the tumor at diagnosis, are shown in Table 2. The results suggested that age, gender, and tumor stage showed no significant differences among the three TME subtypes.

**Table 2:** Clinical characteristics of patients of different subtypes in the LIRI_JP cohort

Variable	C1	C2	C3	*p* value
Gender				0.208
Female	24	11	14	
Male	63	25	65	
Age (years)				0.163
< 60	22	6	11	
≥ 60	65	30	68	
Grade				0.279
I	16	5	9	
II	41	19	37	
III	27	7	26	
IV	3	5	7	

### Transcriptome feature of the TME subtypes

**Fig. 4:**
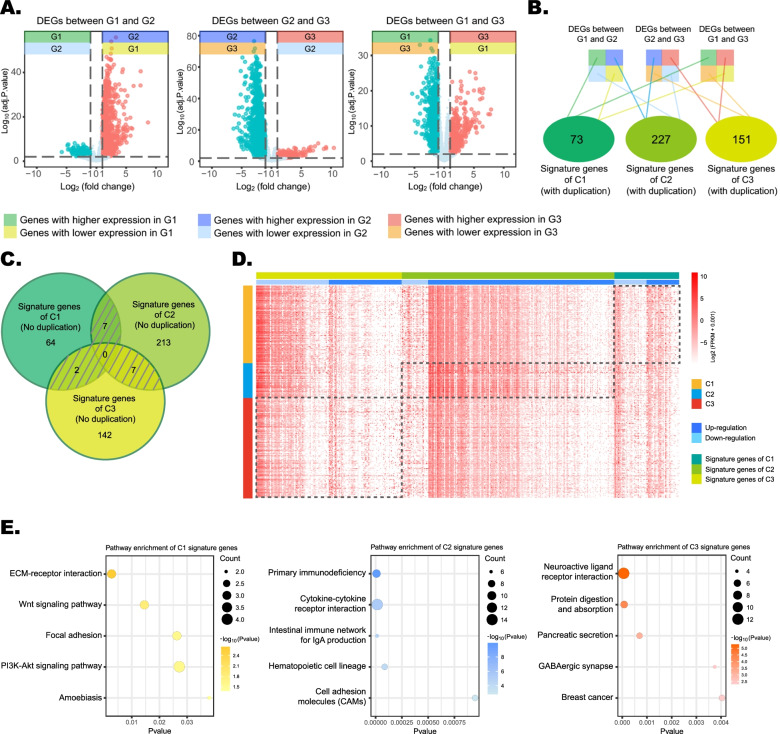
Identification of signature genes of three tumor microenvironment (TME) subtypes and functional annotation. **a** Volcano plots of the differentially expressed genes between C1 and C2, between C2 and C3, and between C1 and C3, respectively. **b** Diagram depicting the process of identification of the signature genes of three TME subtypes. **c** Venn diagram showing the signature genes of three TME subtypes with no duplication. **d** Three different subtypes of signature genes shown as a heatmap. *Abbreviation:* FPKM, fragments per kilobase of exon per million reads mapped. **e** Bubble plots of Kyoto Encyclopedia of Genes and Genomes pathway analysis of TME subtypes’ signature genes

Differential expression analysis was conducted according to the cutoff criteria of an adjusted *p* value of < 0.01 and |log2 (fold-change)| of ≥1. Differentially expressed genes (DEGs) in C1 and C2, C2 and C3, and C1 and C3, respectively, were 400, 781, and 358 (Fig. 4a). Then, after combining the high-expression and low-expression genes and deleting the duplication genes, signature genes were identified, and the numbers of signature genes in C1, C2, and C3 were 64, 213, and 142, respectively (Figs.4b and 4c). A heatmap was then constructed to visualize the expression level of signature genes of C1, C2, and C3 (Fig. 4d).

To explore the functional implications of these signature genes of different subtypes, KEGG pathway enrichment analysis was performed. The results of KEGG enrichment analysis revealed that the signature genes of C1, C2, and C3 were primarily enriched in five KEGG pathways (for C1, extracellular matrix–receptor interaction, the Wnt/PI3K/Akt signaling pathway, focal adhesion, and amoebiasis), 23 pathways (the top five for C2 were primary immunodeficiency, cytokine–cytokine receptor interaction, intestinal immune network for immunoglobulin A production, hematopoietic cell lineage, and cell-adhesion molecules), and 26 pathways (the top five for C3 are neuroactive ligand-receptor interaction, protein digestion and absorption, and pancreatic secretion, GABAergic synapse, and breast cancer), respectively (Table S1). The results are visualized in bubble plots (Fig. 4e). Moreover, we investigated the correlation of immune cells with mutations of *TP53* and *CTNNB1*, which are two representative genes in liver cancer. It was demonstrated that two types of immune cells (fibroblasts and cells of monocytic lineage) have different relative amounts of TME cell populations when comparing between TP53-mutant and TP53 wild-type HCC cells. Analogously, eight immune cells also showed different relative amounts of TME cell populations between CTNBB1-mutant and CTNBB1 wild-type HCC cells. Furthermore, CTNNB1 wild-type HCC cells, compared to CTNBB1-mutant HCC cells, had greater relative amounts of TME cell populations across all eight immune cells (Fig. S2).

### Identification of hub genes of 3 TME subtypes

**Fig. 5:**
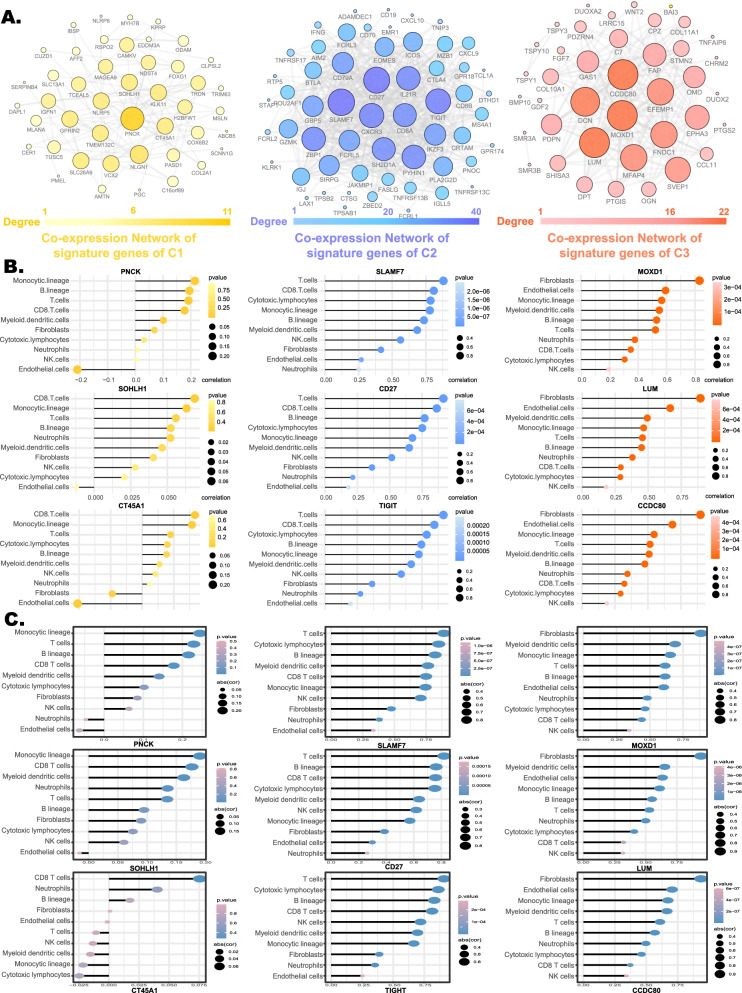
Identification of hub genes from signature genes of different subtypes. **a** Co-expression network of signature genes of different subtypes. The correlation between genes (expression values were log-transformed and verified to be normally distributed) were estimated by Pearson’s correlation analysis. Genes are represented by cycle nodes, whose size represents how many genes are connected to the node, and in gene networks, edges represent interactions between genes. Lollipop plots show the correlation between the expression levels of hub genes and the absolute abundance of TME cell populations in the Cancer Genome Atlas cohort **b** and Liver Cancer-RIKEN,JP cohort **c**. The size of nodes represents the power of the interrelation between genes and TME cell populations, and the shade of nodes represents the significance of the correlation

The signature genes in the three TME subtypes were analyzed and identified by gene co-expression network analysis to determine the core genes for C1, C2, and C3. After excluding the gene pairs with correlation coefficients of < 0.7 in C2 and C3 and those with coefficients of < 0.3 in C1, 45 signature genes for C1, 57 signature genes for C2, and 40 signature genes for C3 were included to construct the co-expression network. Co-expression networks were constructed using Cytoscape (Fig. 5a). Genes were represented by nodes in the network, and interactions between genes were represented by edges.

Network degrees, which describe the number of genes that regulate each other, represented the size and the color shades of the cycle node. Each node in the network represented the number of enriched genes in the gene set, and different colors and sizes represented different degrees of closeness, with the most central genes in the network having the largest degree values. Subsequently, the cytoHubba plugin in Cytoscape was applied to identify hub genes among signature genes of C1 (the top three genes were *PNCK*, *SOHLH1*, and *CT45A1*), C2 (the top three genes were *SLAMF7*, *CD27*, and A T cell immunoreceptor with immunoglobulin and ITIM domains *[TIGIT]*), and C3 (the top three genes were *MOXD1*, *LUM*, and *CCDC80*), respectively. Also, lollipop plots were used to visualize the correlation between hub gene expression and total number of TME cells. The results show that there was a weak correlation between hub genes of C1 and all TME cell populations (correlation coefficient < 0.3), hub genes correlate strongly with C2 and the eight immune cell populations (correlation coefficient > 0.5), and a strong correlation exists between hub genes for C3 and the two stromal cell populations (correlation coefficient > 0.5) (Fig. 5b). This result was reproduced in the LIRI_JP cohort (Fig. 5c).

### Prognostic value of hub genes

The prognosis of the four hub genes was further analyzed and is shown in Fig. S2. The results of survival analysis indicated that all three hub genes of C1 (*PNCK*: HR = 1.84, 95% CI = 1.29–2.64, *p* = 0.001; *SOHLH1*: HR = 1.71, 95% CI = 1.18–2.48, *p* = 0.002; and *CT45A1*: HR = 2.36, 95% CI = 1.27–4.40, *p* < 0.001), two of the three hub genes for C2 (*SLAMF7*: HR = 0.63, 95% CI = 0.42–0.94, *p* = 0.041 and *CD27*: HR = 0.62, 95% CI = 0.42–0.90, *p* = 0.019), and one of the three hub genes for C3 (*MOXD1*: HR = 1.61, 95% CI = 1.09–2.39, *p* = 0.032) had significant prognostic value.

## Discussion

Based on this analysis, three subtypes of immune microenvironment were identified for HCC (C1, C2, and C3). With the highest absolute abundance of TME cell populations and the longest survival time, the potential prognostic value of the PD-1/programmed death ligand 1 (PD-L1) blockade for the C2 patients is worth further investigation. The present analysis showed that patients in C2 had the best prognosis with the largest number of TME cells. This result is in accordance with that of another study on HCC classification, which showed that the patients with the immune-high subtype of TME had a significantly better prognosis [3]. The efficacy of immune checkpoint inhibitors (ICIs) is associated with the level of immune infiltration. Greater infiltration of cytotoxic T-cells is directly related to a better clinical response [4]. It has been demonstrated in previous studies [5–7] that the immune-high subtype correlates with PD-L1 expression in tumor and immune cells (mainly macrophages) and PD1 positivity in CD8^+^ T cells. In clinical trials for HCC, only CTLA-4 and PD-1/PD-L1 inhibitors have been tested to date. In the body, by interacting with PD-1 and PD-L1 on antigen-presenting cells, PD-L1 maintains self-tolerance and inhibits T cell activation [8, 9]. ICIs boost the immune response by depleting the tumor’s cytotoxic T-cells [10]. Hence, it is valuable to study the potential efficacy of ICIs in HCC patients in C2 further.

*CTNNB1* and *TP53* are two major mutation genes in HCC [11]. Previous studies [12] have shown that HCC with a *CTNNB1* mutation is generally well differentiated. As a therapeutic biomarker, therapies targeting *CTNNB1* in HCC have reached promising outcomes [13]. In this analysis, the lowest infiltration of immune cells together with the highest mutations of *CTNNB1* may indicate a lack of response to ICIs. Simultaneously, in this current study, though the TMB in C3 was highest among the three subtypes, this result may not indicate a better response to ICIs [14]. Bagaev et al. [15] previously showed that TMB predicts the immunotherapy effect unsatisfactorily in a pan-cancer analysis (area under the receiver operating characteristic curve [AUC] for TME = 0.82, AUC of TMB = 0.56). An analysis based on TCGA data also found that a higher TMB in HCC is associated with a poorer survival prognosis and negative immune checkpoint activity; the infiltration level of immune cells was higher in the low-TMB group than the high-TMB group, and the heavy mutation load may inhibit immune cell infiltration in HCC. Consistent results were also observed in the C3 group of this study, i.e., a high TMB (Fig. 3b) and a low level of immune cell infiltration (Figs. 2c and 2d). For further verification, we divided patients into two groups according to their level of TMB, and the results showed that the high-TMB group had lower immune cell infiltration (Fig. 3c). This result may contradict the universal notion that high TMB might yield numerous neo-antigens that will activate the anti-tumor immune response, therefore more experiments are necessary.

*FGF19* amplification was found in C2 patients in this analysis, which is reported to be overexpressed in a subtype of HCC patients [16]. *FGF19* signals sent through its receptor, fibroblast growth factor receptor 4 *(FGFR4)*, can induce hepatocyte proliferation as well as glycogen synthesis. *FGFR4* inhibitors, including *BLU-554* and *FGR401*, have been studied as potential treatments for HCC patients that act by interfering with *FGF19*–*FGFR4* signaling and have achieved promising results in phase I and II clinical trials [17]. Hence, further studies in this regard are desirable, as are clinical trials designed to ascertain the therapeutic value of combining immunotherapy and molecular-targeted therapy for C2 patients.

A more thorough understanding of the complex mechanism of TME may stimulate further investigation into combination therapies [18]. The top three hub genes (*PNCK*, *SOHLH1*, and *CT45A1*) identified in C1 correlated with patient prognosis. Human breast cancers overexpress *PNCK* compared to benign breast tissues as *PNCK* plays an important role in mammary development [19]. Furthermore, *CT45A1* overexpression has been confirmed in various cancers with a weak tumorigenic effect [20]. Studies have shown that [21, 22] overexpression of *CT45A1* in breast cancer cells significantly upregulates several oncogenic and metastatic genes, indicating that *CT45A1* may be a promising biomarker for targeted tumor therapy [21].

The top three hub genes (*SLAMF7*, *CD27*, and *TIGIT*) identified in C2 have great potential to be biomarkers of immunotherapy. *SLAMF7* has been regarded previously as a target for immunotherapy in multiple myeloma [23]. CD27 belongs to the TNF (tumor necrosis factor) receptor superfamily and it is expressed exclusively on lymphocytes, and CD27 signaling involving its ligand, CD70, promotes T-cell expansion, survival, and differentiation as well as B-cell and NK cell activation [24]. Varilumab, a CD27 agonist, has already been tested in multiple early-phase clinical trials, either alone or in combination with antiPD-1 therapy. Mouse models treated with CD27 agonists have also revealed these drugs’ preventive role in tumor formation or progression [25]. For cancer immunotherapy, T-cell immunoglobulin and TIGIT, as well as CTLA-4, PD-1, T-cell immunoglobulin 3 and lymphocyte activation gene 3, are the most commonly targeted checkpoints [26]. In melanoma and colon cancer mouse models, anti-*TIGIT* therapy results in tumor regression and improved survival. Although the development of *TIGIT* inhibitors is still in its early phase, several clinical trials of *TIGIT* inhibitors are now ongoing [27]. The synergistic effect of combination therapy involving PD-1 inhibitors and *TIGIT* inhibitors in mouse models suggests that it may be possible to treat lung cancer patients with upregulation of both PD-1 and *TIGIT* [28]. In summary, molecular-targeted therapy alone, immunotherapy alone, or a combination of both may be a potential therapeutic strategy for C2 HCC patients.

Among the top three hub genes detected in C3, the expression of *LUM* was found to be regulated during liver fibrogenesis. Though *LUM* is considered to be essential for hepatic fibrosis, its function in hepatocarcinogenesis has not yet been determined [29, 30]. The signature genes of C1 were primarily enriched in five KEGG pathways (extracellular matrix–receptor interaction, Wnt signaling pathway, Focal adhesion, PI3K–Akt signaling pathway, and amoebiasis). Thus, the therapies targeting molecules in these signaling pathways may be interesting items to explore further in the future.

In general, this study classified the TME of HCC according to varied immune compositions, gene expression levels, and prognoses. On the one hand, it is hoped that this study can develop a deeper understanding of HCC tumor biology and therapeutic response. On the other hand, the new patient-stratification method proposed in this study is expected to provide some guidance for the management of HCC patients in the future and may contribute to precision medicine;however, we still recognize the limitations of our study. To reduce the bias, we analyzed and constructed an immune-stratification model based on the TCGA dataset and validated it in the LIRI_JP dataset. However, all data in this study originate from online datasets, so further experimental validation and patient tumor sample validation are necessary.

## Conclusion

Immunotherapies and/or *FGFR4* inhibitors for HCC patients in C2, *CTNNB1*-targeted therapies for those in C3, and therapies targeting the molecules Wnt–β-catenin pathway and/or the PI3K pathway for those in C1, may be potential therapeutic strategies that warrant further investigation.

## Supplementary Information

**Additional file 1: Table S1.** Results of the Kyoto Encyclopedia of Genes and Genomes enrichment analysis

**Additional file 2: Fig. S1.** Identification of optimal k of the Cancer Genome Atlas. **Fig. S2.** Correlation of the immune cells with *TP53* and *CTNNB1*. *p < 0.05, **p < 0.01, ***p < 0.001, ***p < 0.0001

## Data Availability

The data that support the findings of this study are openly available in the Cancer Genome Atlas database (https://cancergenome.nih.gov) and the International Cancer Genome Consortium portal database (https://dcc.icgc.org/projects/LIRI_JP-JP). The data that support the findings of this study are openly available in the cBioPortal website (http://www.cbioportal.org/index.do). The data that support the findings of this study are openly available in the Enrichr database (http://amp.pharm.mssm.edu/Enrichr/).

## Abbreviations

CTLA-4: Cytotoxic T-lymphocyte-associated protein-4
CTNNB1: Catenin (cadherin-associated protein), β1
DEGs: Differentially expressed genes
FGF19: Fibroblast growth factor-19
FGFR4: Fibroblast growth factor receptor 4
ESTIMATE: Estimation of Stromal and Immune cells in Malignant Tumour Tissues Using Expression Data
AJCC: American Joint Committee on Cancer
HCC: hepatocellular carcinoma
HR: hazard ratio
ICI: Immune checkpoint inhibitor
KEGG: Kyoto Encyclopedia of Genes and Genomes
MST: Median survival time
PD-1: Programmed cell death protein 1
PD-L1: Programmed death ligand 1
TCGA: The Cancer Genome Atlas
TMB: Tumor mutation burden
TME: Tumor microenvironment

## References

[1] Massarweh NN, El-Serag HB (2017). Epidemiology of Hepatocellular Carcinoma and Intrahepatic Cholangiocarcinoma. Cancer Control.

[2] El-Khoueiry AB, Sangro B, Yau T, Crocenzi TS, Kudo M, Hsu C, Kim TY, Choo SP, Trojan J, Welling THR (2017). Nivolumab in patients with advanced hepatocellular carcinoma (CheckMate 040): an open-label, non-comparative, phase 1/2 dose escalation and expansion trial. Lancet (London, England).

[3] Kurebayashi Y, Ojima H, Tsujikawa H, Kubota N, Maehara J, Abe Y, Kitago M, Shinoda M, Kitagawa Y, Sakamoto M (2018). Landscape of immune microenvironment in hepatocellular carcinoma and its additional impact on histological and molecular classification. Hepatology (Baltimore, Md).

[4] Maleki Vareki S (2018). High and low mutational burden tumors versus immunologically hot and cold tumors and response to immune checkpoint inhibitors. Journal for immunotherapy of cancer.

[5] Huang TX, Fu L (2019). The immune landscape of esophageal cancer. Cancer Commun (Lond).

[6] Sharma A, Seow JJW, Dutertre CA, Pai R, Blériot C, Mishra A, Wong RMM, Singh GSN, Sudhagar S, Khalilnezhad S, Erdal S, Teo HM, Khalilnezhad A, Chakarov S, Lim TKH, Fui ACY, Chieh AKW, Chung CP, Bonney GK, Goh BK, Chan JKY, Chow PKH, Ginhoux F, DasGupta R (2020). Onco-fetal reprogramming of endothelial cells drives immunosuppressive macrophages in hepatocellular carcinoma. Cell..

[7] Sun Y, Wu L, Zhong Y, Zhou K, Hou Y, Wang Z, Zhang Z, Xie J, Wang C, Chen D, Huang Y, Wei X, Shi Y, Zhao Z, Li Y, Guo Z, Yu Q, Xu L, Volpe G, Qiu S, Zhou J, Ward C, Sun H, Yin Y, Xu X, Wang X, Esteban MA, Yang H, Wang J, Dean M, Zhang Y, Liu S, Yang X, Fan J (2021). Single-cell landscape of the ecosystem in early-relapse hepatocellular carcinoma. Cell..

[8] Hato T, Goyal L, Greten TF, Duda DG, Zhu AX (2014). Immune checkpoint blockade in hepatocellular carcinoma: current progress and future directions. Hepatology (Baltimore, Md).

[9] Pardoll DM (2012). The blockade of immune checkpoints in cancer immunotherapy. Nat Rev Cancer.

[10] Buonaguro L, Mauriello A, Cavalluzzo B, Petrizzo A, Tagliamonte M (2019). Immunotherapy in hepatocellular carcinoma. Ann Hepatol.

[11] Cancer Genome Atlas Research Network. Electronic address: wheeler@bcm.edu; Cancer Genome Atlas Research Network. Comprehensive and Integrative Genomic Characterization of Hepatocellular Carcinoma. Cell. 2017 Jun 15;169(7):1327–1341.e23. doi: 10.1016/j.cell.2017.05.046. PMID: 28622513; PMCID: PMC5680778. 10.1016/j.cell.2017.05.046 PMC5680778 28622513

[12] Chen M, Zhang B, Topatana W, Cao J, Zhu H, Juengpanich S, Mao Q, Yu H, Cai X (2020). Classification and mutation prediction based on histopathology H&E images in liver cancer using deep learning. NPJ Precis Oncol.

[13] Schulze K, Imbeaud S, Letouze E, Alexandrov LB, Calderaro J, Rebouissou S, Couchy G, Meiller C, Shinde J, Soysouvanh F (2015). Exome sequencing of hepatocellular carcinomas identifies new mutational signatures and potential therapeutic targets. Nat Genet.

[14] Yarchoan M, Hopkins A, Jaffee EM (2017). Tumor mutational burden and response rate to PD-1 inhibition. N Engl J Med.

[15] Bagaev A, Kotlov N, Nomie K, Svekolkin V, Gafurov A, Isaeva O, Osokin N, Kozlov I, Frenkel F, Gancharova O, Almog N, Tsiper M, Ataullakhanov R, Fowler N (2021). Conserved pan-cancer microenvironment subtypes predict response to immunotherapy. Cancer Cell.

[16] Dogan-Topal B, Li W, Schinkel AH, Beijnen JH, Sparidans RW (2019). Quantification of FGFR4 inhibitor BLU-554 in mouse plasma and tissue homogenates using liquid chromatography-tandem mass spectrometry. J Chromatogr B Anal Technol Biomed Life Sci.

[17] Katoh M (2016). FGFR inhibitors: effects on cancer cells, tumor microenvironment and whole-body homeostasis (review). Int J Mol Med.

[18] Tian M, Shi Y, Liu W, Fan J. Immunotherapy of hepatocellular carcinoma: strategies for combinatorial intervention. Sci China Life Sci. 2019. 10.1007/s11427-018-9446-2 31119560

[19] Gardner HP, Ha SI, Reynolds C, Chodosh LA (2000). The caM kinase, Pnck, is spatially and temporally regulated during murine mammary gland development and may identify an epithelial cell subtype involved in breast cancer. Cancer Res.

[20] Tang F, Tang S, Guo X, Yang C, Jia K (2017). CT45A1 siRNA silencing suppresses the proliferation, metastasis and invasion of lung cancer cells by downregulating the ERK/CREB signaling pathway. Mol Med Rep.

[21] Shang B, Gao A, Pan Y, Zhang G, Tu J, Zhou Y, Yang P, Cao Z, Wei Q, Ding Y, Zhang J, Zhao Y, Zhou Q (2014). CT45A1 acts as a new proto-oncogene to trigger tumorigenesis and cancer metastasis. Cell Death Dis.

[22] Chen YT, Hsu M, Lee P, Shin SJ, Mhawech-Fauceglia P, Odunsi K, Altorki NK, Song CJ, Jin BQ, Simpson AJ, Old LJ (2009). Cancer/testis antigen CT45: analysis of mRNA and protein expression in human cancer. Int J Cancer.

[23] van Driel BJ, Liao G, Engel P, Terhorst C. Responses to microbial challenges by SLAMF receptors. Front Immunol 2016 Jan 20;7:4. doi: 10.3389/fimmu.2016.00004. PMID: 26834746; PMCID: PMC4718992. 10.3389/fimmu.2016.00004 PMC4718992 26834746

[24] Burugu S, Dancsok AR, Nielsen TO (2018). Emerging targets in cancer immunotherapy. Semin Cancer Biol.

[25] Sakanishi T, Yagita H (2010). Anti-tumor effects of depleting and non-depleting anti-CD27 monoclonal antibodies in immune-competent mice. Biochem Biophys Res Commun.

[26] Chiu DK, Yuen VW, Cheu JW, Wei LL, Ting V, Fehlings M, Sumatoh H, Nardin A, Newell EW, Ng IO, Yau TC, Wong CM, Wong CC. Hepatocellular carcinoma cells up-regulate PVRL1, stabilizing PVR and inhibiting the cytotoxic T-cell response via TIGIT to mediate tumor resistance to PD1 inhibitors in mice. Gastroenterology 2020 Aug;159(2):609–623. 10.1053/j.gastro.2020.03.074. Epub 2020 Apr 8. PMID: 32275969. 10.1053/j.gastro.2020.03.074 32275969

[27] Rotte A, Jin JY, Lemaire V (2018). Mechanistic overview of immune checkpoints to support the rational design of their combinations in cancer immunotherapy. Annals Oncol: official journal of the European Society for Medical Oncology.

[28] Wang Q, Chen X, Hay N (2017). Akt as a target for cancer therapy: more is not always better (lessons from studies in mice). Br J Cancer.

[29] Baghy K, Tatrai P, Regos E, Kovalszky I (2016). Proteoglycans in liver cancer. World J Gastroenterol.

[30] Charlton M, Viker K, Krishnan A, Sanderson S, Veldt B, Kaalsbeek AJ, Kendrick M, Thompson G, Que F, Swain J, Sarr M (2009). Differential expression of lumican and fatty acid binding protein-1: new insights into the histologic spectrum of nonalcoholic fatty liver disease. Hepatology..

