# Supplementary figures and images for: A stratification model of hepatocellular carcinoma based on expression profiles of cells in the tumor microenvironment
Source: BMC Cancer. 2022 Jun 4;22:613. doi: 10.1186/s12885-022-09647-5 (PMC9167552; doi:10.1186/s12885-022-09647-5)

# K-means consensus clustering

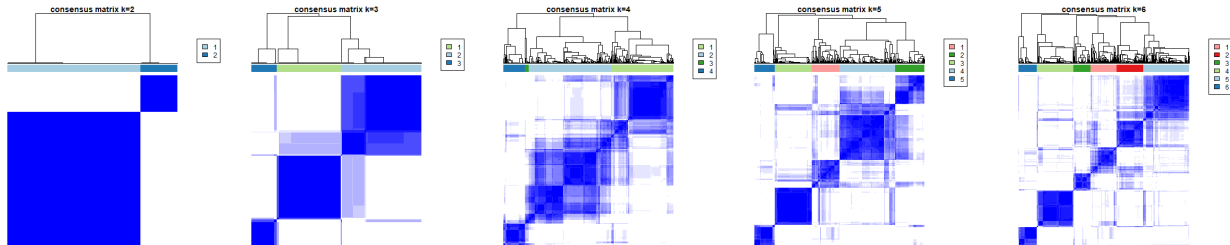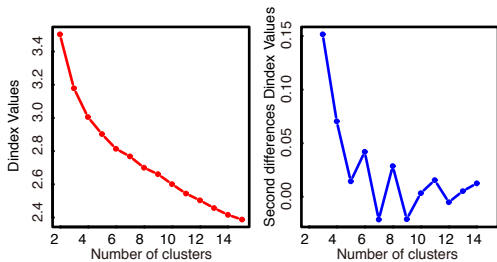

Supplement: Supplementary file 2 — Additional file 2: Fig. S1. Identification of optimal k of the Cancer Genome Atlas. Fig. S2. Correlation of the immune cells with TP53 and CTNNB1. *p < 0.05, **p < 0.01, ***p < 0.001, ***p < 0.0001 [file 12885_2022_9647_MOESM2_ESM.pdf]
